# Diabetes duration-specific association of dietary inflammatory index with the risk of mortality among individuals with diabetes

**DOI:** 10.1186/s13098-025-01771-z

**Published:** 2025-06-24

**Authors:** Xi Chen, Lixia Lin, Yuhe Tan, Ya Zhu, Wuxing Song, Hao Wang, Xufang Sun

**Affiliations:** 1 Department of Ophthalmology, Hubei Key Laboratory of Otolaryngologic and Ophthalmic Diseases, Tongji Hospital, Tongji Medical College, Huazhong University of Science and Technology, Wuhan, China; 2 School of Physical Education and Health, Hubei University of Chinese Medicine, Hubei Shizhen Laboratory, Wuhan, China; 3 Key Laboratory of Environment and Health, State Key Laboratory of Environmental Health (Incubating), School of Public Health, Tongji Medical College, Ministry of Education, Huazhong University of Science and Technology, Wuhan, China; 4 Department of Epidemiology and Biostatistics, Ministry of Education Key Laboratory of Environment and Health, School of Public Health, Tongji Medical College, Huazhong University of Science and Technology, Wuhan, China

**Keywords:** Dietary inflammatory index, Diabetes duration, Diabetes, Mortality, Cohort study

## Abstract

**Background:**

The dietary inflammatory index (DII) is a literature-derived index to assess the inflammatory potential of diet. While numerous studies have linked a higher DII score to an increased risk of mortality, there are limited studies among individuals with diabetes.

**Objective:**

To investigate the association of the DII with all-cause mortality, cardiovascular disease (CVD) mortality, and cancer mortality among individuals with diabetes, and to explore whether diabetes duration could modify these associations.

**Methods:**

A total of 9942 participants with diabetes from the UK Biobank were included. The DII scores were calculated based on 24-h dietary data. Outcomes were ascertained from linked records. The hazard ratios (HRs) and 95% confidence intervals (CIs) for mortality according to DII quartiles were estimated using Cox proportional hazards models.

**Results:**

During the median follow-up period of 12.1 years, 1225 (12.3%) participants with diabetes died. The multivariable HRs (95% CIs) in the highest quartile compared with the lowest DII quartiles were 1.30 (1.06, 1.58) for mortality, 1.67 (1.13, 2.48) for CVD mortality, and 1.00 (0.73, 1.38) for cancer mortality. Spline regression analysis indicated significant nonlinear associations between DII and mortality, while the relationship with CVD mortality was linear. Moreover, diabetes duration significantly modified the association between DII and all-cause mortality (*P*-interaction = 0.002). In participants with diabetes duration of less than 5 years, the HR (95% CIs) for all-cause mortality in the highest versus lowest DII quartile was 1.73 (1.25, 2.39), while for those with diabetes duration of 5 years or more, the HR (95% CIs) was 1.08 (0.83, 1.39).

**Conclusion:**

A higher DII, indicating a pro-inflammation dietary pattern, is associated with an elevated risk of all-cause mortality and CVD mortality in individuals with diabetes. Notably, the association with mortality is more pronounced in those with diabetes duration less than 5 years.

**Supplementary Information:**

The online version contains supplementary material available at 10.1186/s13098-025-01771-z.

## Introduction

Diabetes has become a major global health threat, with an estimated 529 million individuals affected worldwide in 2021, corresponding to an age-standardized prevalence of 6.1% [1]. Individuals with diabetes are at an increased risk of premature mortality due to cardiovascular diseases (CVD), cancer, and non-cardiovascular noncancer causes [2]. Globally, diabetes-related deaths account for 35.5% of deaths from non-communicable diseases and 2.7% of deaths from all causes [3]. Given this substantial burden, managing key risk factors is crucial for reducing diabetes-related mortality. Among these, diet, as a modifiable lifestyle factor, plays an important role in the prevention of mortality in individuals with diabetes [4].

Growing evidence implicates diabetes in the dysregulation of inflammatory cytokines, which may contribute to mortality in this population [5]. Various dietary ingredients possess anti-inflammatory properties, including vitamin C and E, as well as a range of bioactive compounds such as polyphenols, flavonoids, carotenoids, etc [6, 7]. Because of the complex interactions among these ingredients, examining overall dietary quality rather than individual nutrients has garnered increasing attention. The dietary inflammation index (DII) quantifies the inflammatory potential of diet with a composite index, based on an extensive literature search incorporating cell culture, animal, and epidemiologic studies on the effect of inflammatory makers (i.e., C-reactive protein, anti-inflammatory cytokines, and tumor necrosis factor-α) [8, 9]. DII represents a complicated set of exposures which often interact, and whose cumulative effect modifies both inflammatory responses and health outcomes. Previous research has indicated associations between DII scores and all-cause as well as cause-specific mortality in general population [10–15]. Compared with individuals without diabetes, those diagnosed diabetes had higher DII score, indicating a higher dietary inflammatory potential [13]. However, in the diabetic population, results on the association between DII and mortality are lacking and controversial. Although a few studies have reported a positive association of DII with all-cause mortality among diabetic population, these studies were limited from rather short follow-up periods (less than 5 years) [16, 17]. Additionally, because of the small size of individuals with diabetes, other studies found no significant associations [14, 18]. Furthermore, research on the link between DII and cause-specific mortality, such as CVD or cancer mortality, in patients with diabetes is limited and inconclusive.

In addition, disease duration has been recognized as an important determinant in identifying the prognosis of diabetes [19]. Evidence suggests individuals with a shorter duration of diabetes (less than 5 years) may benefit more from intensive glycemic control than those with longer disease duration [20]. These findings suggest that tailoring the management of diabetes according to disease duration may be more effective. Longer disease duration has been reported to be linked to higher levels of inflammatory biomarkers in patients with diabetes due to persistent hyperglycemia and vascular damage [21]. Also, risk of mortality in adults with diabetes increase with longer duration of disease [22]. However, the potential modifying effect of diabetes duration when examining the association between DII and mortality risk has not yet been identified, which may have substantial public health implications on translating epidemiological findings to meaningful public health actions.

Therefore, the present study aimed to investigate the association of DII with mortality, CVD mortality, and cancer mortality among patients with diabetes within a large prospective cohort study. Importantly, we also examined the potential modifying effect of diabetes duration on these associations to provide a more nuanced understanding of the relationship between dietary inflammation and morality risk in this population.

## Methods

### Study population

The UK Biobank is a population-based prospective cohort study encompassing over 500 000 individuals aged between 37 and 73 years at enrollment, representing 5.5% of those invited to participant [23]. Briefly, the study recruited participants in the United Kingdom from 22 assessment centers across Scotland, England and Wales between 2006 and 2010. Participants completed a touch screen questionnaire, engaged in a face-to-face interview, underwent a series of physical measurements, and provided biological samples at baseline. Ethical approval for the UK Biobank was granted by the North West Research Ethics Committee (06/MRE08/65), and all participants provided formed consent. This research was conducted using the UK Biobank Resource under Application Number 88,159.

In the present study, we restricted our research to 30,455 participants with diabetes. Diabetes at baseline was identified using UK Biobank algorithms developed by Eastwood et al. through self-reported medical history, use of oral hypoglycemic medication or insulin, and biochemical examination for glycated hemoglobin A1c (HbA1c) [24]. We used the cutoffs of HbA1c for defining diabetes, 48 mmol/mol (6.5%). The exclusion criteria were as follows: (1) missing data on age at diabetes diagnosis, (2) incomplete information on 24-h dietary assessments, and (3) implausible energy intake (men with < 800 kcal/d or > 4200 kcal/d; women with < 600 kcal/d or > 3500 kcal/d) [25]. The rationale for excluding participants who reported implausible energy intake was based on potential confounding or bias associated with extreme energy intake. Finally, 9942 participants were included in the present study (Figure S1).

### Dietary inflammatory index

Dietary intake was assessed using the Oxford WebQ, a web-based 24-h tool designed to record the consumption of 206 food items and 32 beverages during the past 24 h [26]. The assessment tool inquired about the previous day’s consumption with questions as: “Did you have any of these yesterday?” or “How much of the following did you drink yesterday?” Energy and nutrient intake were quantified based on McCane and Widdowson’s The Composition of Food, 5th edition [27]. Dietary intakes were averaged for participants who had more than one 24-hour dietary recall.

The dietary inflammatory index (DII) is a population-based tool derived from the literature, designed to measure the inflammatory potential of the diet [9]. The original DII encompasses 45 food parameters, including macronutrients, micronutrients, bioactive compounds, and tea that are linked to inflammatory markers, e.g., C-reactive protein, IL-1β, IL4, IL-6, IL-10, and tumor necrosis factor-α. Food parameters receive a score based on their impact on these markers, with + 1 for pro-inflammation effects, -1 for anti-inflammatory effects, and 0 for neutral effect. Scores are weighted according to the type and number of foods in the target literature [28]. Global mean values and standard deviations for 11 food parameters were created from databases across different countries, from which z-scores and central percentiles for each participant’s food parameters are calculated [9]. The food parameter specific DII score is calculated by multiplying the central percentile of each food parameter and its corresponding “food parameter-specific overall inflammatory effects scores”. Finally, the total DII score is obtained by adding all the “food parameter-specific overall inflammatory effects scores”. In the present study, the food or nutrient parameters used to calculate DII were excluded under the following conditions according to the criteria described in a previous study [28]: (1) information on foods or nutrients was not collected or provided, (2) specific intake data were not provided, and (3) units of intake provided could not be directly used to calculate DII. Ultimately, 29 eligible food or nutrient parameters were identified in the UK Biobank (Table S1).

### All-cause mortality and cause-specific mortality

The primary outcomes were all-cause mortality, CVD mortality, and cancer mortality. Mortality information was derived from death certificates by the National Health Service (NHS), Information Center (England and Wales), and the NHS Central Register Scotland (Scotland), with records available until December 31th, 2021. According to the International Classification of Diseases, 10th revision (ICD-10), deaths from CVD were coded as I00-I99, and deaths from cancer were coded as C00-C97. Person-years were calculated from the attending date to the occurrence of death, loss to follow-up, or the end date of follow-up (December 31th, 2021).

### Covariates

A touchscreen questionnaire was employed to obtain information on baseline characteristics and lifestyle factors including age, sex (men or women), race/ethnicity (white or non-white), education level (college or others), smoking status (never, former, or current), and drinking status (never, former, or current). Physical activity was categorized as adequate if participants met the highest tertile of metabolic equivalent tase (MET) minutes per week. The Townsend Deprivation Index was derived from participants’ zip codes of residence, using aggregated data on automobiles, unemployment, homeownership, and household overcrowding. Participants’ height and weight were measured, and body mass index (BMI) was calculated as weight in kilograms divided by the square of height in meters. Total energy intake was determined from 24-h dietary recall assessments. The answer of the question “What was your age when the diabetes was first diagnosed?” was used to ascertain the age of diabetes onset, and diabetes duration at baseline was calculated accordingly.

### Statistical analysis

Baseline characteristics are presented as mean and standard deviation (SD) or frequency (%) across the overall population and according to DII quartiles. Individuals were divided into four categories based on the DII quartiles, with quartile 1 as the reference group, and Cox proportional hazards models were used to estimate hazard ratios (HRs) and 95% confidence intervals (CIs) for all-cause mortality, CVD mortality, and cancer mortality across DII quartiles. DII was also treated as a continuous variable to assess the HRs (95% CIs) associated with each one-point increase in the DII. The median DII value for each quartile was used as a continuous variable in the Cox proportional hazards model to evaluate the linear trend across quartiles. For missing data, we included a missing category for categorical variables and used mean value imputation for missing data on continuous variables. The percentage of missing values are present in Table S2.

Three models were built. Model 1 was adjusted for age at recruitment (years) and sex (men or women), and total energy intake (kcal/d); Model 2 was further adjusted for race/ethnicity (white or non-white), education (college or others), Townsend Deprivation Index, drinking (never, former, or current), smoking (never, former, or current), BMI (kg/m^2^), physical activity (adequate or not); Model 3 was further adjusted for diabetes duration (years).

To test the consistency of the results, we further investigated the association of DII and the outcomes of interest, stratifying by age (< 60 or ≥ 60 years), BMI (< 30 or ≥ 30 kg/m^2^), sex (men or women), education (college or others), race/ethnicity (white or non-white), and diabetes duration (< 5 or ≥ 5 years) [19]. Interactions between these stratification variables and the DII were estimated using the likelihood ratio test. To explore potential nonlinear trends of the association, a restricted cubic spline (RCS) model was applied, with knots at the 25th, 50th, and 75th percentiles of the DII distribution; the reference value (HR = 1) set at the 10th percentile.

Several sensitivity analyses were conducted to test the robustness of our results. First, we repeated the analyses after excluding participants with missing values. Secondly, we adjusted for the time interval between baseline and completion of 24-hour dietary recalls. For participants with more than one 24-hour dietary recall, the time interval was averaged as time intervals between baseline recruitment and each dietary recall. Thirly, HbA1c (a stable indicator for glycemic control), use of antidiabetic drugs, and use of antihypertensive drugs were further adjusted for. Fourth, the analyses were performed using multiple imputation by chained equations with 5 imputations (SAS PROC MI with a fully conditional specification method and PROC MIANALYZE) for participants who had missing data.

All statistical analyses were conducted using SAS software version 9.4 (SAS Institute, Cary, NC, USA) and R software (version 4.2.2). A two-sided *P* value < 0.05 was determined to be significant.

## Results

A total of 9942 participants were included in the present study, with a median follow-up duration of 12.1 years. During follow-up, 1225 (12.3%) deaths occurred, including 323 (3.3%) caused by CVD and 498 (5.0%) caused by cancer. DII scores ranged from − 5.75 to 4.86. Participants’ characteristics were described in Table 1. Individuals with diabetes with higher DII scores were more likely to be women, younger, less educated, less physically active, current smokers, use antihypertensive drugs, and with higher BMI and lower total energy intake.

**Fig. 1:**
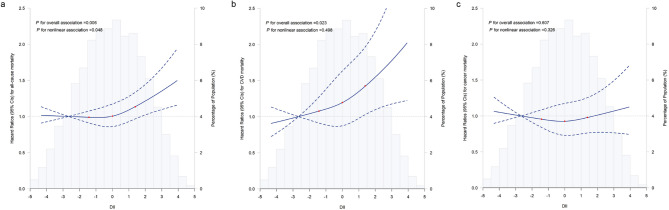
Restricted cubic spline for testing the hypothesis of nonlinear correlation between (**a**) all-cause mortality, (**b**) CVD mortality, (**c**) cancer mortality and DII. Knots were placed at the 25th, 50th, and 75th percentiles of the DII score, and the reference value was set the 10th percentile. Spline curves represent hazard ratios (HRs) adjusted for age at recruitment (continuous, years), sex (men, women), race/ethnicity (White, non-White), education (college or university degree, others), total energy intake (continuous, kcal/d), Townsend Deprivation Index (continuous), drinking (never, former, current), smoking (never, former, current), BMI (continuous, kg/m^2^), physical activity (adequate, inadequate), and diabetes duration (continuous, years)

The associations of the DII and mortality are shown in Table 2. DII was positively associated with mortality across model 1 to model 3 (*P* < 0.05). In comparisons with participants with the first quartile of DII, participants with the highest quartile of DII had a multivariable-adjusted HR of 1.30 (95% CI 1.06, 1.58) for all-cause mortality (*P* for trend = 0.006), 1.67 (95% CI 1.13, 2.48) for CVD mortality (*P* for trend = 0.006), and 1.00 (95% CI 0.73, 1.38) for cancer mortality (*P* for trend = 0.879). For each unit increment in DII score, there was a 6% higher risk of all-cause mortality (HR: 1.06, 95% CI: 1.02–1.11) and a 13% higher risk of CVD mortality (HR: 1.13, 95% CI: 1.04–1.21). Spline regression analysis indicated a significant nonlinear association between DII and mortality (*p* = 0.006, *p*-value for nonlinearlity = 0.048, Fig. 1-a), while the association between DII and CVD mortality was linear (*p* < 0.023, *p*-value for nonlinearlity = 0.498; Fig. 1-b). No significant association was observed of cancer mortality related to DII (Fig. 1-c).

**Fig. 2:**
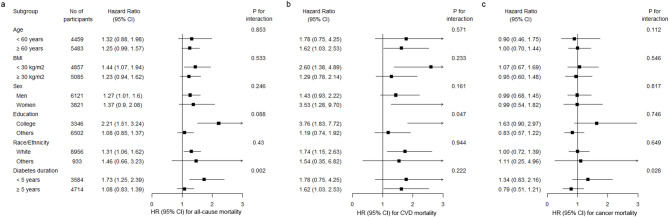
Associations between DII and (**a**) all-cause mortality, (**b**) CVD mortality, and (**c**) cancer mortality across subgroups. Hazard ratio for the fourth quartile compared with the first quartile of DII. Adjusted for age at recruitment (continuous, years), sex (men, women), race/ethnicity (White, non-White), education (college or university degree, others), total energy intake (continuous, kcal/d), Townsend Deprivation Index (continuous), drinking (never, former, current), smoking (never, former, current), BMI (continuous, kg/m^2^), physical activity (adequate, inadequate), and diabetes duration (continuous, years)

Stratified analyses revealed that the positive association between DII and all-cause mortality consistent across subgroups defined by age, BMI, sex, education, and ethnicity (Fig. 2, Table S3-S5). The association was more pronounced among participants with diabetes duration of less than 5 years compared to those with 5 or more years (*P* for interaction = 0.002 for all-cause mortality, *P* for interaction = 0.028 for cancer mortality). Comparing the highest quartile of the DII to the lowest quartile, the HRs (95% Cs) were 1.73 (1.25, 2.39) for all-cause mortality and 1.34 (0.83, 2.16) for cancer mortality among those with diabetes duration less than 5 years, and 1.08 (0.83, 1.39) for all-cause mortality and 0.79 (0.51, 1.21) for cancer mortality among those with diabetes duration more than 5 years. For CVD mortality, the association between DII and CVD mortality was more pronounced among participants higher educated (*P* for interaction = 0.047), while no statistically significant interaction was observed between DII and diabetes duration.

In the sensitivity analyses, the results were generally robust when excluding participants with missing values, adjusting for the time interval between baseline and completion of 24-hour dietary recalls, adjusting for HbA1c, use of antidiabetic drugs, and use of antihypertensive drugs, or using multiple imputation methods (Table S6-S9).

**Table 1:** Baseline characteristics according to quartiles of dietary inflammatory index (DII) among diabetic patients in the UK biobank study (*N* = 9942)

Characteristics	Overall	DII				*P* value
		First quartile	Second quartile	Third quartile	Fourth quartile	
Number	9942	2485	2486	2486	2485	
Age, years	58.9 ± 7.2	59.4 ± 7.1	59.3 ± 7.0	58.7 ± 7.1	58.2 ± 7.6	< 0.001
Men	6121 (61.6)	1603 (64.5)	1583 (63.7)	1498 (60.3)	1437 (57.8)	< 0.001
Education, college or university degree^*^	3346 (33.7)	861 (34.7)	919 (37.0)	824 (33.2)	741 (29.8)	< 0.001
Townsend deprivation index^*^	-1.0 ± 3.1	-1.2 ± 3.1	-1.2 ± 3.0	-1.1 ± 3.1	-0.5 ± 3.3	< 0.001
Race/ethnicity, white^*^	8956 (90.1)	2252 (90.6)	2307 (92.8)	2260 (90.9)	2137 (86.0)	< 0.001
Adequate physical activity	3314 (33.3)	984 (39.6)	847 (34.1)	780 (31.4)	703 (28.3)	< 0.001
BMI, kg/m^2 *^	31.0 ± 5.9	30.5 ± 5.7	30.7 ± 5.9	31.1 ± 5.8	31.7 ± 6.1	< 0.001
Smoking status^*^						< 0.001
Never	4551 (45.8)	1104 (44.4)	1142 (45.9)	1155 (46.5)	1150 (46.3)	
Former	4483 (45.1)	1196 (48.1)	1148 (46.2)	1099 (44.2)	1040 (41.9)	
Current	866 (8.7)	174 (7.0)	186 (7.5)	222 (8.9)	284 (11.4)	
Drinking status^*^						< 0.001
Never	566 (5.7)	126 (5.1)	113 (4.6)	132 (5.3)	195 (7.9)	
Former	574 (5.8)	137 (5.5)	128 (5.2)	132 (5.3)	177 (7.1)	
Current	8792 (88.4)	2216 (89.2)	2244 (90.3)	2220 (89.3)	2112 (85.0)	
Use of antidiabetic drugs	6011 (60.5)	1452 (58.4)	1505 (60.5)	965 (61.2)	533 (61.7)	0.094
Use of antihypertensive drugs	5994 (60.3)	1436 (57.8)	1519 (61.1)	1534 (61.7)	1505 (60.6)	0.025
Total Energy intake, kcal/d	2036 ± 576	2438 ± 571	2168 ± 485	1930 ± 443	1606 ± 441	< 0.001
HbA1c, mmol/mol^*^	52.4 ± 13.5	52.1 ± 13.5	52.5 ± 13.7	52.3 ± 12.9	52.8 ± 14.0	0.337
Diabetes duration	9.3 ± 11.9	8.3 ± 12.0	9.2 ± 11.6	9.0 ± 11.3	9.5 ± 12.8	0.562

Data was presented as mean ± SD or n (%).

*P* values for differences in baseline characteristics were estimated by ANOVA or chi-squared test.

^*^The missing values of these covariates ranged from 0.1 to 6.2%.

BMI, body mass index. HbA1c, glycated hemoglobin A1c

**Table 2:** Multivariable-adjusted hrs (95% CIs) for the associations of DII with mortality among diabetic patients (*n* = 9942)

	DII				*P* for Trend	Continuous Analysis, per 1 unit increase
	First quartile	Second quartile	Third quartile	Fourth quartile		
All-cause mortality						
Cases/person-years	300/29,525	284/29,685	307/29,583	334/29,243		
Model 1	1.00 (reference)	0.99 (0.84, 1.17)	1.17 (0.98, 1.39)	1.40 (1.16, 1.68)	< 0.001	1.08 (1.05, 1.12)
Model 2	1.00 (reference)	0.97 (0.82, 1.15)	1.10 (0.93, 1.31)	1.21 (1.01, 1.46)	0.024	1.05 (1.01, 1.09)
Model 3	1.00 (reference)	0.99 (0.83, 1.18)	1.14 (0.95, 1.37)	1.30 (1.06, 1.58)	0.006	1.06 (1.02, 1.11)
CVD Mortality						
Cases/person-years	73/29,525	76/29,685	82/29,583	92/29,243		
Model 1	1.00 (reference)	1.14 (0.82, 1.58)	1.39 (0.99, 1.96)	1.79 (1.24, 2.58)	0.001	1.14 (1.07, 1.23)
Model 2	1.00 (reference)	1.13 (0.81, 1.57)	1.31 (0.93, 1.84)	1.53 (1.06, 2.21)	0.018	1.10 (1.03, 1.18)
Model 3	1.00 (reference)	1.15 (0.81, 1.65)	1.48 (1.03, 2.12)	1.67 (1.13, 2.48)	0.006	1.13 (1.04, 1.21)
Cancer Mortality						
Cases/person-years	132/29,525	119/29,685	125/29,583	122/29,243		
Model 1	1.00 (reference)	0.92 (0.71, 1.19)	1.04 (0.80, 1.35)	1.08 (0.81, 1.45)	0.493	1.04 (0.98, 1.09)
Model 2	1.00 (reference)	0.90 (0.70, 1.15)	0.97 (0.75, 1.27)	0.96 (0.71, 1.28)	0.878	1.01 (0.95, 1.06)
Model 3	1.00 (reference)	0.89 (0.68, 1.17)	0.99 (0.74, 1.32)	1.00 (0.73, 1.38)	0.879	1.02 (0.96, 1.09)

Model 1 was adjusted for age at recruitment (continuous, years), sex (men, women), and total energy intake (continuous, kcal/d).

Model 2 was further adjusted for race/ethnicity (White, non-White), education (college or university degree, others), Townsend Deprivation Index (continuous), drinking (never, former, current), smoking (never, former, current), BMI (continuous, kg/m^2^), physical activity (adequate, inadequate).

Model 3 was further adjusted for diabetes duration (continuous, years)

## Discussion

In our study, among individuals with diabetes, the DII is found to be positively associated with the risk of mortality and CVD mortality, but not cancer mortality. Moreover, the association of the DII with mortality is more pronounced among those with diabetes duration of less than 5 years. These findings suggest that for diabetic patients, pro-inflammation diet is supposed to be avoided to prevent an increased risk of mortality, and the early stage of diabetes (duration less than 5 years) but not late stage (duration of 5 years or more) may be the critical window for effective diet intervention.

Previous studies have found that individuals with a higher DII score were at a higher risk of all-cause mortality, CVD mortality, and certain cancer mortality in general population [10–14, 18]. While the present study extends this finding to individuals with diabetes based on a large prospective cohort. Consistent with our findings, some studies have reported that a higher DII is associated with an increased risk of all-cause mortality and CVD mortality among patients with diabetes [16, 17], but they were limited from rather short follow-up periods with less than 5 years and did not explore the association between DII and cancer mortality. In contrast, there were two studies based on National Health and Nutrition Examination Survey (NHANES) which failed to observe the association between DII and mortality in diabetic population [14, 18]. The discrepancies in the findings may be attributed to genetic predispositions, lifestyle, and dietary habits variations among the different populations, and the limited statistical power due to the relatively small sample size of individuals with diabetes (from 968 to 4951) in these studies. Based on longer follow-up duration (more than 12 years) and a much larger sample size (more than 9900), we promoted other researchers’ studies and provided more credible results. Additionally, sensitivity analyses excluding missing covariate data or using multiple imputation for missing data did not alter our findings.

Due to that the course of the disease is an important factor affecting the prognosis of diabetes, the present study further evaluated the association of the DII with outcomes of interest separately among individuals with short or long diabetes duration. A significant positive association of the DII with mortality was found among patients with diabetes duration of less than 5 years, but not among patients with diabetes duration of 5 years or more, which has not been reported before. These findings highlight that the early 5 years of new-onset diabetes is a critical window for preventing premature death, and healthcare professionals can recommend patients with new-onset diabetes to adopt an anti-inflammatory diet, to manage the risk of premature death. Whereas, from the perspective of preventing premature death, patients with diabetes of 5 years or more are less likely to benefit from adopting an anti-inflammatory diet. Similarly, previous evidence regarding metformin, which has been reported to have anti-inflammatory effects, partly supports the finding of the present study [29]. A meta-analysis encompassing 13 studies on mortality, most of which featured follow-up duration of less than 5 years, suggested that individuals with diabetes taking metformin had a lower risk of mortality compared to those without diabetes [30]. Conversely, another study involving 3234 individuals at high risk of type 2 diabetes, with a followed-up duration over 20 years, failed to demonstrate a decreased risk of mortality associated with metformin use [31]. This further supports the notion that interventions early in the disease course may have a more substantial impact on survival.

The positive association between the DII and mortality and CVD mortality among diabetic patients may be explained by several mechanisms. Diabetic patients generally exhibit chronic low-grade inflammation, and a high DII diet can exacerbate this inflammatory state, leading to endothelial dysfunction, atherosclerosis, and thrombosis, thereby increasing the risk of CVD [32–34]. Also, high DII diets are typically rich in high-calorie foods, refined carbohydrates, and unhealthy fats, which can elevate oxidative stress and metabolic disturbances, further activating inflammatory pathways and promoting cardiovascular damage [4, 35, 36]. Additionally, a high DII diet may exacerbate insulin resistance, a critical risk factor common to both diabetes and CVD, thereby potentially increasing the risk of CVD mortality through mechanisms such as poor glycemic control, hypertension, and dyslipidemia [37–39].

Some limitations of the present study need comments as well. Firstly, as an observational study, the present study cannot establish a causal relationship between DII and mortality. Secondly, the identified associations of the present study may be subject to residual confounding by unmeasured variables, although some important covariates have been considered. Additionally, the dietary intake was self-reported, which may introduce recall bias. Also, some dietary components such as caffeine, onion, garlic, eugenol, ginger, onion, turmeric were not included in the calculation of the DII. However, these missing variables are less likely to drastically affect the potential of the DII to predict inflammation, and it has been shown that the DII’s ability to predict inflammation remains when the number of food variables is reduced from 45 to 23 [8]. Nevertheless, we recognize that including these additional components could potentially enhance the sensitivity and specificity of the DII as a marker of diet-related inflammation. Future studies may consider incorporating these components to further optimize the predictive model of the DII. Additionally, future studies could examine the impact of specific anti-inflammatory dietary components, the role of diabetes treatments with anti-inflammatory properties, and intervention trials targeting diet early in diabetes.

In conclusion, using data from a large perspective cohort, the current study found that a higher DII score, indicated a pro-inflammatory diet, was associated with higher risk of mortality and CVD mortality among population with diabetes, especially among those with diabetes duration of less than 5 years. These findings highlighted the importance of early screening for diabetes and suggested that treatment of anti-inflammatory diet should be added in clinical guidelines soon after diabetes diagnosis, which may have important implications to reduce the mortality burden associated with diabetes of shorter duration.

## Electronic supplementary material

Below is the link to the electronic supplementary material.

Supplementary Material 1

## Data Availability

The data that support the findings of this study are available from UK Biobank but restrictions apply to the availability of these data, which were used under license for the current study, and so are not publicly available. Data are however available from the authors upon reasonable request and with permission of UK Biobank.
