# Supplementary material for: Diabetes duration-specific association of dietary inflammatory index with the risk of mortality among individuals with diabetes
Source: Diabetol Metab Syndr. 2025 Jun 24;17:243. doi: 10.1186/s13098-025-01771-z (PMC12186389; doi:10.1186/s13098-025-01771-z)
Supplement: Supplementary file 1 — Supplementary Material 1 [file 13098_2025_1771_MOESM1_ESM.docx]

Supplementary Materials

**Diabetes duration-specific association of dietary inflammatory index with the risk of mortality among individuals with diabetes**

Xi Chen, ^1#^ Lixia Lin, ^2#^ Yuhe Tan,^1^ Ya Zhu, ^3^ Wuxing Song, ^3^ Hao Wang ^3*^ Xufang Sun, ^1*^

502401 participants recruited to the UK Biobank between 2006 and 2010

471946 without diabetes at baseline excluded

30455 patients with diabetes

20513 excluded

- 20266 patients without complete information on 24-h dietary assessments
- 247 who had unreasonable energy intake (man with < 800 kcal/d or > 4200 kcal/d or female with < 600 kcal/d or > 3500 kcal/d

9942 patients included

**Figure S1** Flowchart of the participants in the study.

**Table S1** Food patterns used in this study for calculating the dietary inflammatory index, and their respective inflammatory effect scores.

|  | Food parameter | Parameter-specific inflammatory effect score* |
| --- | --- | --- |
|  | Energy (kcal/day) | 0.180 |
| Macronutrients | Alcohol (g/day) | -0.278 |
|  | Carbohydrate (g/day) | 0.097 |
|  | Total Protein (g/day) | 0.021 |
|  | Total fat (g/day) | 0.298 |
|  | Polyunsaturated fat (g/day) | -0.337 |
|  | Saturated fat (g/day) | 0.373 |
|  | Monounsaturated fat (g/day) | -0.009 |
|  | Trans fatty acid (g/day) | 0.229 |
|  | ω-3 fatty acids (g/day) | -0.436 |
|  | ω-6 fatty acids (g/day) | -0.159 |
|  | Dietary fiber (g/day) | -0.663 |
| Bioactive compounds | Cholesterol (mg/day) | 0.110 |
|  | β-Carotene (μg/day) | -0.584 |
| Micronutrients | Vitamin B12 (μg/day) | 0.106 |
|  | Vitamin B6 (mg/day) | -0.365 |
|  | Niacin (mg/day) | -0.246 |
|  | Selenium (μg/day) | -0.191 |
|  | Thiamin (mg/day) | -0.098 |
|  | Riboflavin (mg/day) | -0.068 |
|  | Vitamin A (RE/day) | -0.401 |
|  | Vitamin C (mg/day) | -0.424 |
|  | Vitamin D (mcg/day) | -0.446 |
|  | Vitamin E (mg/day) | -0.419 |
|  | Folate (μg/day) | -0.190 |
|  | Fe (mg/day) | 0.032 |
|  | Mg (mg/day) | -0.484 |
|  | Zn (mg/day) | -0.313 |
| Others | Tea (g/day) | -0.536 |

*Positive scores indicated that the corresponding food parameters were associated with a pro-inflammatory effect, while negative scores indicated that the corresponding food parameters were associated with anti-inflammatory effect.

**Table S2** Percentage of missing values of the covariates

| **Covariates** | **Missing percentage** |
| --- | --- |
| Race/Ethnicity | 0.5% |
| Townsend deprivation index | 0.2% |
| BMI | 0.5% |
| Education | 0.9% |
| HbA1c, mmol/mol | 6.2% |
| Smoking | 0.4% |
| Drinking | 0.1% |

**Table S3** Stratified analyses for the association of DII with mortality among diabetic patients.

|  |  | DII | | | | *P* for Interaction |
| --- | --- | --- | --- | --- | --- | --- |
|  | n | First quartile | Second quartile | Third quartile | Fourth quartile |  |
| Age |  |  |  |  |  | 0.853 |
| < 60 years | 4459 | 1.00 (reference) | 1.23 (0.85, 1.77) | 1.11 (0.76, 1.63) | 1.32 (0.88, 1.98) |  |
| ≥ 60 years | 5483 | 1.00 (reference) | 0.91 (0.74, 1.12) | 1.12 (0.9, 1.38) | 1.25 (0.99, 1.57) |  |
| BMI |  |  |  |  |  | 0.533 |
| < 30 kg/m^2^ | 4857 | 1.00 (reference) | 0.91 (0.7, 1.18) | 1.12 (0.85, 1.47) | 1.44 (1.07, 1.94) |  |
| ≥ 30 kg/m^2^ | 5085 | 1.00 (reference) | 1.09 (0.85, 1.4) | 1.18 (0.92, 1.52) | 1.23 (0.94, 1.62) |  |
| Sex |  |  |  |  |  | 0.246 |
| Men | 6121 | 1.00 (reference) | 0.95 (0.77, 1.16) | 1.16 (0.94, 1.42) | 1.27 (1.01, 1.6) |  |
| Women | 3821 | 1.00 (reference) | 1.19 (0.8, 1.76) | 1.12 (0.75, 1.68) | 1.37 (0.9, 2.08) |  |
| Education |  |  |  |  |  | 0.088 |
| College | 3346 | 1.00 (reference) | 1.11 (0.8, 1.54) | 1.4 (0.98, 2) | 2.21 (1.51, 3.24) |  |
| Others | 6502 | 1.00 (reference) | 0.95 (0.77, 1.18) | 1.07 (0.86, 1.33) | 1.08 (0.85, 1.37) |  |
| Race/Ethnicity |  |  |  |  |  | 0.430 |
| White | 8956 | 1.00 (reference) | 1.03 (0.86, 1.24) | 1.16 (0.96, 1.4) | 1.31 (1.06, 1.62) |  |
| Others | 933 | 1.00 (reference) | 0.56 (0.22, 1.4) | 1.23 (0.56, 2.68) | 1.46 (0.66, 3.23) |  |
| Diabetes duration |  |  |  |  |  | 0.002 |
| < 5 years | 3584 | 1.00 (reference) | 0.98 (0.72, 1.32) | 1.19 (0.88, 1.62) | 1.73 (1.25, 2.39) |  |
| ≥ 5 years | 4714 | 1.00 (reference) | 0.98 (0.79, 1.23) | 1.1 (0.88, 1.39) | 1.08 (0.83, 1.39) |  |

Adjusted for age at recruitment (continuous, years), sex (men, women), race/ethnicity (White, non-White), education (college or university degree, others), total energy intake (continuous, kcal/d), Townsend Deprivation Index (continuous), drinking (never, former, current), smoking (never, former, current), BMI (continuous, kg/m^2^), physical activity (adequate, inadequate), and diabetes duration (continuous, years).

**Table S4** Stratified analyses for the association of DII with CVD mortality among diabetic patients.

|  |  | DII | | | | *P* for Interaction |
| --- | --- | --- | --- | --- | --- | --- |
|  | n | First quartile | Second quartile | Third quartile | Fourth quartile |  |
| Age |  |  |  |  |  | 0.571 |
| < 60 years | 4459 | 1.00 (reference) | 1.71 (0.78, 3.77) | 1.9 (0.86, 4.2) | 1.78 (0.75, 4.25) |  |
| ≥ 60 years | 5483 | 1.00 (reference) | 1.03 (0.69, 1.53) | 1.35 (0.89, 2.03) | 1.62 (1.03, 2.53) |  |
| BMI |  |  |  |  |  | 0.233 |
| < 30 kg/m^2^ | 4857 | 1.00 (reference) | 1.4 (0.8, 2.43) | 1.65 (0.91, 2.99) | 2.6 (1.38, 4.89) |  |
| ≥ 30 kg/m^2^ | 5085 | 1.00 (reference) | 1.04 (0.66, 1.65) | 1.4 (0.89, 2.2) | 1.29 (0.78, 2.14) |  |
| Sex |  |  |  |  |  | 0.161 |
| Men | 6121 | 1.00 (reference) | 1.06 (0.72, 1.56) | 1.38 (0.93, 2.05) | 1.43 (0.93, 2.22) |  |
| Women | 3821 | 1.00 (reference) | 1.87 (0.69, 5.04) | 2.28 (0.84, 6.2) | 3.53 (1.28, 9.7) |  |
| Education |  |  |  |  |  | 0.047 |
| College | 3346 | 1.00 (reference) | 1.26 (0.65, 2.44) | 1.77 (0.88, 3.57) | 3.76 (1.83, 7.72) |  |
| Others | 6502 | 1.00 (reference) | 1.1 (0.72, 1.68) | 1.39 (0.91, 2.12) | 1.19 (0.74, 1.92) |  |
| Race/Ethnicity |  |  |  |  |  | 0.944 |
| White | 8956 | 1.00 (reference) | 1.2 (0.83, 1.73) | 1.53 (1.05, 2.24) | 1.74 (1.15, 2.63) |  |
| Others | 933 | 1.00 (reference) | 1.22 (0.29, 5.16) | 1.72 (0.42, 6.98) | 1.54 (0.35, 6.82) |  |
| Diabetes duration |  |  |  |  |  | 0.222 |
| < 5 years | 3584 | 1.00 (reference) | 1.71 (0.78, 3.77) | 1.9 (0.86, 4.2) | 1.78 (0.75, 4.25) |  |
| ≥ 5 years | 4714 | 1.00 (reference) | 1.03 (0.69, 1.53) | 1.35 (0.89, 2.03) | 1.62 (1.03, 2.53) |  |

Adjusted for age at recruitment (continuous, years), sex (men, women), race/ethnicity (White, non-White), education (college or university degree, others), total energy intake (continuous, kcal/d), Townsend Deprivation Index (continuous), drinking (never, former, current), smoking (never, former, current), BMI (continuous, kg/m^2^), physical activity (adequate, inadequate), and diabetes duration (continuous, years).

**Table S5** Stratified analyses for the association of DII with cancer mortality among diabetic patients.

|  |  | DII | | | | *P* for Interaction |
| --- | --- | --- | --- | --- | --- | --- |
|  | n | First quartile | Second quartile | Third quartile | Fourth quartile |  |
| Age |  |  |  |  |  | 0.112 |
| < 60 years | 4459 | 1.00 (reference) | 1.02 (0.59, 1.79) | 0.67 (0.35, 1.26) | 0.9 (0.46, 1.75) |  |
| ≥ 60 years | 5483 | 1.00 (reference) | 0.83 (0.6, 1.14) | 1.05 (0.76, 1.45) | 1 (0.7, 1.44) |  |
| BMI |  |  |  |  |  | 0.546 |
| < 30 kg/m^2^ | 4857 | 1.00 (reference) | 0.79 (0.53, 1.16) | 1.13 (0.76, 1.67) | 1.07 (0.67, 1.69) |  |
| ≥ 30 kg/m^2^ | 5085 | 1.00 (reference) | 1.03 (0.69, 1.54) | 0.9 (0.59, 1.37) | 0.95 (0.6, 1.48) |  |
| Sex |  |  |  |  |  | 0.817 |
| Men | 6121 | 1.00 (reference) | 0.84 (0.6, 1.16) | 1.06 (0.77, 1.48) | 0.99 (0.68, 1.45) |  |
| Women | 3821 | 1.00 (reference) | 1.08 (0.63, 1.85) | 0.82 (0.46, 1.48) | 0.99 (0.54, 1.82) |  |
| Education |  |  |  |  |  | 0.746 |
| College | 3346 | 1.00 (reference) | 1.11 (0.69, 1.8) | 1.47 (0.87, 2.46) | 1.63 (0.9, 2.97) |  |
| Others | 6502 | 1.00 (reference) | 0.81 (0.58, 1.14) | 0.85 (0.6, 1.2) | 0.83 (0.57, 1.22) |  |
| Race/Ethnicity |  |  |  |  |  | 0.649 |
| White | 8956 | 1.00 (reference) | 0.93 (0.7, 1.23) | 0.98 (0.73, 1.31) | 1 (0.72, 1.39) |  |
| Others | 933 | 1.00 (reference) | - | 1.65 (0.45, 6.01) | 1.11 (0.25, 4.96) |  |
| Diabetes duration |  |  |  |  |  | 0.028 |
| < 5 years | 3584 | 1.00 (reference) | 0.84 (0.54, 1.31) | 1.12 (0.72, 1.74) | 1.34 (0.83, 2.16) |  |
| ≥ 5 years | 4714 | 1.00 (reference) | 0.91 (0.64, 1.3) | 0.91 (0.62, 1.33) | 0.79 (0.51, 1.21) |  |

Adjusted for age at recruitment (continuous, years), sex (men, women), race/ethnicity (White, non-White), education (college or university degree, others), total energy intake (continuous, kcal/d), Townsend Deprivation Index (continuous), drinking (never, former, current), smoking (never, former, current), BMI (continuous, kg/m^2^), physical activity (adequate, inadequate), and diabetes duration (continuous, years).

**Table S6** Sensitivity analyses of association between DII and mortality after excluding participants with missing values.

|  | DII | | | | *P* for Trend | Continuous Analysis, per 1 score increase |
| --- | --- | --- | --- | --- | --- | --- |
|  | First quartile | Second quartile | Third quartile | Fourth quartile |  |  |
| N | 2485 | 2486 | 2486 | 2485 |  |  |
| Mortality | 1.00 (reference) | 1 (0.84, 1.19) | 1.16 (0.97, 1.39) | 1.32 (1.08, 1.61) | 0.003 | 1.07 (1.03, 1.11) |
| CVD Mortality | 1.00 (reference) | 1.17 (0.82, 1.66) | 1.5 (1.05, 2.15) | 1.7 (1.15, 2.52) | 0.004 | 1.13 (1.05, 1.22) |
| Cancer Mortality | 1.00 (reference) | 0.9 (0.68, 1.19) | 1.01 (0.76, 1.35) | 1.02 (0.74, 1.41) | 0.767 | 1.03 (0.97, 1.09) |

Adjusted for age at recruitment (continuous, years), sex (men, women), race/ethnicity (White, non-White), education (college or university degree, others), total energy intake (continuous, kcal/d), Townsend Deprivation Index (continuous), drinking (never, former, current), smoking (never, former, current), BMI (continuous, kg/m^2^), physical activity (adequate, inadequate), diabetes duration (continuous, years).

**Table S7** Sensitivity analyses of association between DII and mortality adjusted for the time interval between baseline and completion of 24-hour dietary recalls.

|  | DII | | | | *P* for Trend | Continuous Analysis, per 1 score increase |
| --- | --- | --- | --- | --- | --- | --- |
|  | First quartile | Second quartile | Third quartile | Fourth quartile |  |  |
| N | 2485 | 2486 | 2486 | 2485 |  |  |
| Mortality | 1.00 (reference) | 1.00 (0.83, 1.19) | 1.15 (0.96, 1.38) | 1.29 (1.06, 1.58) | 0.006 | 1.06 (1.02, 1.10) |
| CVD Mortality | 1.00 (reference) | 1.16 (0.81, 1.65) | 1.49 (1.04, 2.14) | 1.66 (1.12, 2.46) | 0.006 | 1.12 (1.04, 1.21) |
| Cancer Mortality | 1.00 (reference) | 0.90 (0.68, 1.18) | 1.00 (0.75, 1.33) | 1.00 (0.73, 1.38) | 0.873 | 1.02 (0.96, 1.08) |

Adjusted for age at recruitment (continuous, years), sex (men, women), race/ethnicity (White, non-White), education (college or university degree, others), total energy intake (continuous, kcal/d), Townsend Deprivation Index (continuous), drinking (never, former, current), smoking (never, former, current), BMI (continuous, kg/m^2^), physical activity (adequate, inadequate), diabetes duration (continuous, years), and time interval between baseline and completion of 24-hour dietary recalls (continuous, years).

**Table S8** Sensitivity analyses of association between DII and mortality adjusted for HbA1c, use of antidiabetic drugs, and use of antihypertensive drugs.

|  | DII | | | | *P* for Trend | Continuous Analysis, per 1 score increase |
| --- | --- | --- | --- | --- | --- | --- |
|  | First quartile | Second quartile | Third quartile | Fourth quartile |  |  |
| N | 2485 | 2486 | 2486 | 2485 |  |  |
| Mortality | 1.00 (reference) | 0.97 (0.81, 1.16) | 1.13 (0.94, 1.36) | 1.27 (1.04, 1.56) | 0.008 | 1.06 (1.02, 1.10) |
| CVD Mortality | 1.00 (reference) | 1.12 (0.79, 1.6) | 1.45 (1.01, 2.08) | 1.65 (1.11, 2.45) | 0.007 | 1.12 (1.04, 1.21) |
| Cancer Mortality | 1.00 (reference) | 0.88 (0.67, 1.16) | 1.00 (0.75, 1.33) | 0.99 (0.72, 1.37) | 0.875 | 1.02 (0.96, 1.08) |

Adjusted for age at recruitment (continuous, years), sex (men, women), race/ethnicity (White, non-White), education (college or university degree, others), total energy intake (continuous, kcal/d), Townsend Deprivation Index (continuous), drinking (never, former, current), smoking (never, former, current), BMI (continuous, kg/m^2^), physical activity (adequate, inadequate), diabetes duration (continuous, years), HbA1c (continuous, mmol/mol), use of antidiabetic drugs (yes, no), use of antihypertensive drugs (yes, no).

**Table S9** Sensitivity analyses of association between DII and mortality using multiple imputation by chained equations for continuous variables.

|  | DII | | | | *P* for Trend | Continuous Analysis, per 1 score increase |
| --- | --- | --- | --- | --- | --- | --- |
|  | First quartile | Second quartile | Third quartile | Fourth quartile |  |  |
| N | 2485 | 2486 | 2486 | 2485 |  |  |
| Mortality | 1.00 (reference) | 0.98 (0.82, 1.17) | 1.15 (0.96, 1.38) | 1.30 (1.06, 1.59) | 0.001 | 1.06 (1.02, 1.11) |
| CVD Mortality | 1.00 (reference) | 1.13 (0.79, 1.61) | 1.48 (1.03, 2.13) | 1.68 (1.13, 2.50) | 0.001 | 1.12 (1.04, 1.22) |
| Cancer Mortality | 1.00 (reference) | 0.88 (0.67, 1.17) | 1.02 (0.77, 1.37) | 1.01 (0.73, 1.39) | 0.470 | 1.01 (0.94, 1.08) |

Adjusted for age at recruitment (continuous, years), sex (men, women), race/ethnicity (White, non-White), education (college or university degree, others), total energy intake (continuous, kcal/d), Townsend Deprivation Index (continuous), drinking (never, former, current), smoking (never, former, current), BMI (continuous, kg/m^2^), physical activity (adequate, inadequate), and diabetes duration (continuous, years).
